# Structural characteristics of a mitochondrial control region from *Myotis* bat (Vespertilionidae) mitogenomes based on sequence datasets

**DOI:** 10.1016/j.dib.2019.103830

**Published:** 2019-03-26

**Authors:** Md M. Rahman, Kwang B. Yoon, Yung C. Park

**Affiliations:** a Dept. of Biotechnology and Genetic Engineering, Islamic University, Kushtia, Bangladesh; b Division of Forest Science, Kangwon National University, Chuncheon 24341, Republic of Korea; c Inje County Office (186), Environment Protection Division, Inje-ro, Inje-eup, Inje-gun, Gangwon-do, Republic of Korea

**Keywords:** *Myotis*, Control region, Tandem repeat sequence, CSB, ETAS

## Abstract

The datasets included sequences of a control region from *Myotis* bat mitogenomes. The control region (1706–2005 bp) of the *Myotis* mitogenomes was divided into three domains similar to that of other mammals, which included the common conserved blocks (ETAS domain, Central domain, and CSB domain). Several long tandem repeat sequences were present between the upstream of control regions and ETAS1. The size, base composition, and copy number of the long tandem repeat sequences differed between the *Myotis* species. Short tandem repeat sequences were also found between CSB1 and CSB2 in the CSB domain.

**Table:** Specification table

Subject area	Biology
More specific subject area	Evolutionary Biology
Type of data	Table, figure, and word file
How data was acquired	Downloaded from NCBI GenBank
Data format	Analyzed DNA sequence
Experimental factors	Alignment of *Myotis* control region with Clustal W implemented in Geneious Pro 5.5.9, Tandem Repeats Finder program [1]
Experimental features	Features of domains and repeated sequences of the mitochondrial control region of the genus *Myotis* bats
Data source location	*Myotis formosus*[2] and *M. macrodactylus*[3] samples were collected in South Korea, *M. muricola*[4] in Malaysia, and *M. davidii*[5] and *M. brandtii*[6] in China
Data accessibility	*M. muricola:*https://www.ncbi.nlm.nih.gov/nuccore/NC_029422.1?from=15460&to=17224&report=fasta *M. davidii:*https://www.ncbi.nlm.nih.gov/nuccore/NC_025568.1?from=15461&to=17464&report=fasta *M. brandii:*https://www.ncbi.nlm.nih.gov/nuccore/NC_025308.1?from=15451&to=17400&report=fasta *M. formosus:*https://www.ncbi.nlm.nih.gov/nuccore/HQ184048.1?from=15454&to=17159&report=fasta *M. macrodactylus:*https://www.ncbi.nlm.nih.gov/nuccore/KF440685.1?from=15462&to=17466&report=fasta
Related research article	F. Liu, Y. Song, S.Yan, J. Luo, F. Jiang, Structure and sequence variation of the mitochondrial DNA control region in *Myotis macrodactylus*, Chin. J. Zoo*.* 44 (2009) 19–27.

**Table:** 

**Value of the data** • These data will provide fundamental information to future molecular evolutionary studies of *Myotis* bats. • The data will contribute to understanding rapid evolution in control regions of mammalian mitogenomes. • The characteristics of the primary sequence of the control region will provide valuable information on population genetics, phylogeny, and phylogeography, which would be helpful in decision-making in bat ecological control and management programs.

## 1 Data

**Fig. 1:**
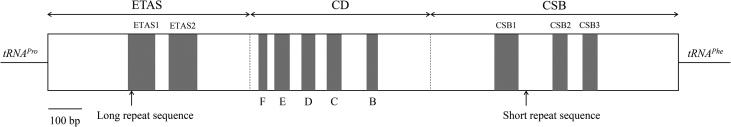
Schematic diagram of the mitochondrial control region (CR) of the genus *Myotis*. The CR consists of three domains (ETAS, CD, and CSB). Highly conserved blocks were present in ETAS1-2, F—B, and CSB1-3 of the three domains. Long tandem repeats were present between CR upstream and ETAS1 and short tandem repeats were present between CSB1 and CSB2. Each copy of long and short repeat was marked in the figure.

**Fig. 2:**
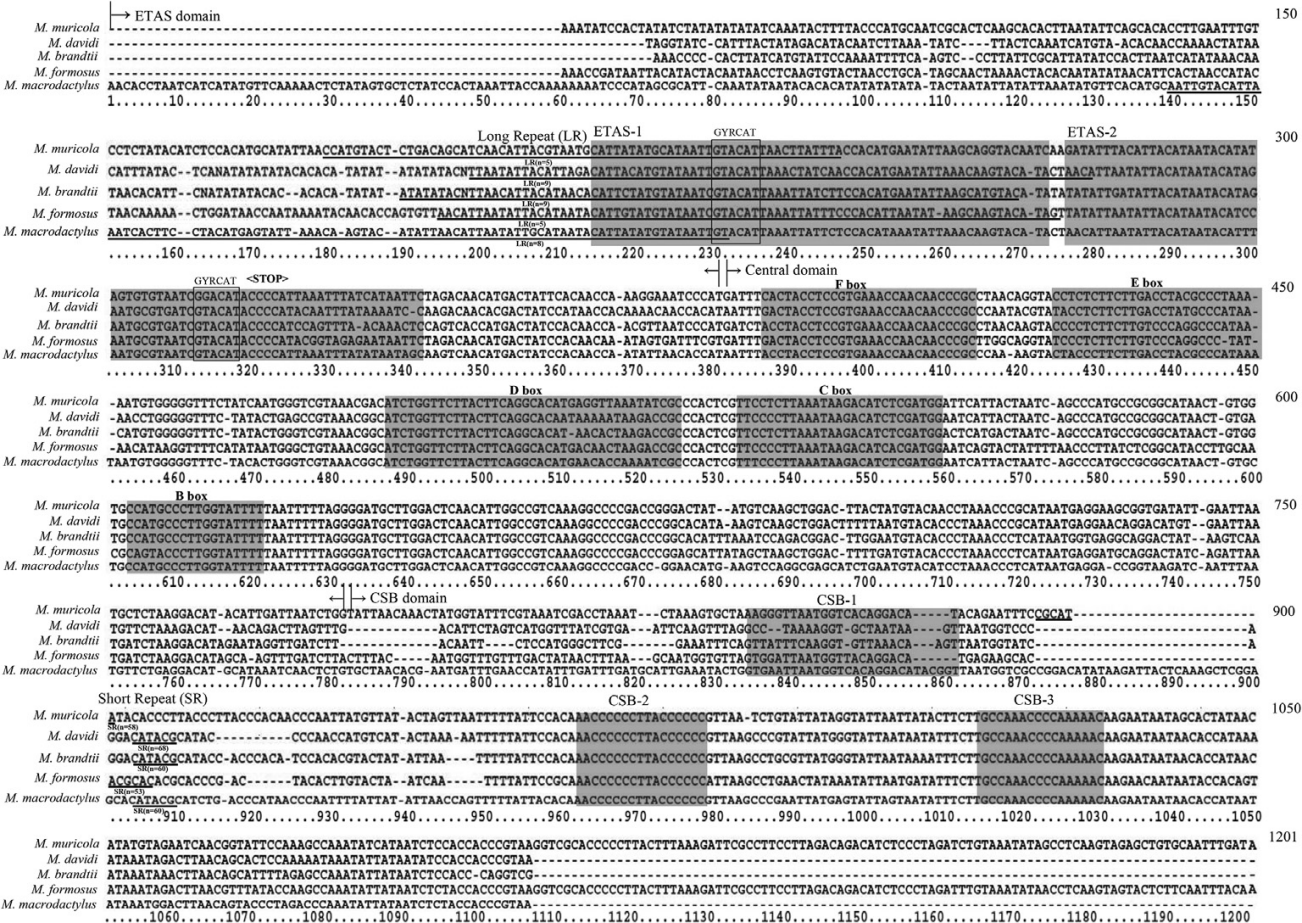
Dataset of the aligned CR sequences of *Myotis* bats. The datasets include only one copy of the tandem repeat sequences, which were underlined and indicated as long and short repeat sequences. The shaded areas indicate highly conserved sequences of ETAS1-2 blocks within ETAS, F-B blocks within the CD, and CSB1-3 blocks within CSB. The putative point of arrest of replication is indicated as (<STOP>). Regions of two GYRCAT (Y=C or T, R = A or G) motifs are indicated as boxes.

The mitochondrial control region (CR), which is the most rapidly evolving region of the mitochondrial DNA, is one of the most commonly used molecular markers in population genetics and phylogenetic studies. We reported the structural characteristics of the CR from the genus *Myotis*, such as tandem repeat sequences and sequence composition, by analyzing the sequences extracted from five whole genomes. These characteristics may be responsible for the fast evolution of CR [2], [3], [4], [5], [6]. The datasets of CR sequences showed three common domains: extended terminal associated sequences (ETAS), central domain (CD), and conserved sequence block (CSB) (Fig. 1). Several copies of long tandem repeat sequences were located between the CR upstream and ETAS1 with the final copy positioned on ETAS1 (Fig. 1). Several short tandem repeat sequences were located between CSB1 and CSB2 (Fig. 2). The conserved sequence motifs (GYRCAT) were present in both regions of ETAS1 and ETAS2 within the ETAS domain. Highly conserved sequences were also found in the five regions of F to B boxes in the CD, and the three regions of CSB1 to CSB3 in the CSB domain (Fig. 2).

## 2 Experimental design, materials, and methods

### 2.1 Sequence data collection

Whole mitogenome sequences of five species of the genus *Myotis* (*M. muricola*, *M. brandtii*, *M. formosus*, *M. macrodactylus*, and *M. davidii*) have been previously reported [2], [3], [4], [5], [6]. We extracted the CR sequences of these five mitogenomes from GenBank (Table 1, Table 2).

**Table 1:** Tandem repeat sequences in mitochondrial control region of Myotis bats.

Species (Accession no.)	Long repeat sequence				Short repeat sequence			References for whole mitogenome sequences
	Motif sequence	Length of a repeat sequence (bp)	Copy number	Total length of long repeat sequence region (bp)	Motif sequence	Copy number	Total length of short repeat sequence region (bp)	
M. muricola (KT213444)	CCACATGAATATTAAGCAAGTACTTTAACAACATTAATATTACATAATACATTATATGTATAATTGTACATTAACTTATTTA	82	5.3	433	CGCATA	58.2	349	[4]
M. davidii (KM233172)	TTAATATTACATTAGACATTACATGTATAATTGTACATTAAACTATCAACCACATGAATATTAAACAAGTACATACTAACA	81	9.1	741	CATACG	68.8	413	[5]
M. brandtii (KM199849)	ATATATATATTAACATTACATAACACATTCTATGTATAATCGTACATTAAATTATCTTCCACATGAATATTAAGCATGTAC	81	9.3	757	CATACG	60.8	365	[6]
M. formosus (HQ184048)	ATTAATATTACATAATACATTGTATGTATAATCGTACATTAAATTATTTCCCACATTAATATAAGCAAGTACATAGTTAT	80	5.3	420	ACGCAT	53.8	323	[2]
M. macrodactylus (KF440685)	AATTGTACATTAAATTATTTTCCACATGAATATTAAACAAGTACATACTAACATTAATATTACATAATACATTATATGTAT	81	8.9	721	CATACG	60.5	363	[3]

**Table 2:** Size (bp) of three domains in mitochondrial control region of five Myotis bats including each of one copy of long and short repeat sequences.

Species	ETAS	CD	CSB	Total size
*M. muricola*	317	392	388	1097
*M. davidii*	289	400	261	950
M. brandtii	293	392	263	948
*M. formosus*	313	395	365	1073
M. macrodactylus	368	393	323	1084
Mean ± SD	316 ± 31.5	394.4 ± 3.4	320 ± 57.9	1030.4 ± 74.8

### 2.2 Processing and analysis of mitochondrial control region sequences of Myotis bats

#### 2.2.1 Sequence and primary structure of the mitochondrial control region

The CR sequences from *Myotis* bats were aligned using Clustal W implemented in Geneious Pro 5.5.9 (Auckland, New Zealand) and edited with BioEdit 7.02 (Tom Hall, USA). The CR sequences were annotated and characterized using other mammalian CR sequences [7], [8], [9], [10], [11], [12] as references. The size of the CR sequences ranged from 1706 (*M. formosus*) to 2005 bp (*M. mcrodactylus*) in length. The CR sequences were subdivided into three main domains: ETAS, CD, and CSB (Fig. 1).

#### 2.2.2 Detection and analysis of tandem repeat sequences

The tandem repeat sequences in the CR region were investigated using Tandem Repeats Finder program [1], and we discovered the motif of the tandem repeat sequences, length of repeats, and copy number. Two type of tandem repeat sequences were found in the CR. Tandem repeats with long sequence motifs were present between CR upstream and ETAS-1 in the ETAS domain, and tandem repeats with short sequence motifs were present between CSB1 and CSB-2 (Fig. 1 and Table 2). The size of the long repeat sequence ranged from 80 bp in *M. formosus* to 82 bp in *M. muricola* and the copy number ranged from 5.3 copies in *M. muricola* and *M. formosus* to 9.3 copies in *M. brandtii*. The size of the short repeat sequence was 6 bp in all CRs, and the copy number ranged from 53.8 copies in *M. formosus* to 68.8 copies in *M. davidii* (Table 1). Excluding these tandem repeat regions, except for each copy of the long and short repeat motifs, the size of the CR ranged from 948 bp in *M. brandtii* to 1097 bp in *M. muricola* (Table 2).

#### 2.2.3 Detection and analysis of the conserved region

We discovered sequence-conserved regions through the mitochondrial CRs based on comparative analysis of the datasets. The conserved sequences identified within the domains were in ETAS1-2, F—B boxes, and CSB1-3 (Fig. 2). ETAS1 was more conserved than ETAS2, suggesting that the former might be functionally more important in mtDNA replication than ETAS2 [7], [8], [13], [14]. The conserved sequence motif ‘GYRCAT’ was present in two locations within ETAS: one of them within ETAS1 and the other within ETAS2 (Fig. 2). The putative point of arrest of D-loop synthesis, ‘ACCCC’, was situated within ETAS2, next to the 3′ end of GYRCAT (Fig. 2). Similarly, putative points of arrest of D-loop synthesis were proposed to be ACCCC in rhinolophid bats [10] and ACCCC, GCCCC, or TCCCC in leaf-nosed bats [11].
