# Supplementary material for: Structural characteristics of a mitochondrial control region from Myotis bat (Vespertilionidae) mitogenomes based on sequence datasets
Source: Data Brief. 2019 Mar 26;24:103830. doi: 10.1016/j.dib.2019.103830 (PMC6477160; doi:10.1016/j.dib.2019.103830)
Supplement: Multimedia component 1 [file mmc1.doc]

Conflict of Interest and Authorship Conformation Form

Please check the following as appropriate:

- All authors have participated in (a) conception and design, or analysis and interpretation of the data; (b) drafting the article or revising it critically for important intellectual content; and (c) approval of the final version.
- This manuscript has not been submitted to, nor is under review at, another journal or other publishing venue.
- The authors have no affiliation with any organization with a direct or indirect financial interest in the subject matter discussed in the manuscript
- The following authors have affiliations with organizations with direct or indirect financial interest in the subject matter discussed in the manuscript:

Author’s name Affiliation

**Rahman Md Mafizur** : *Dept. of Biotechnology and Genetic engineering, Islamic University, Kushtia*

**Kwang bae Yoon**: *Inje county office (186), Environment Protection Division, Inje-ro, Inje-eup, Inje-gun, Gangwon-do, Republic of Korea*

**Yung Chul, Park :** *Division of Forest Science, Kangwon National University, Chuncheon 24341, Republic of Korea*
